# The genome sequence of Goldilocks Aster, *Galatella linosyris* (L. Rchb.f., 1853) (Asterales: Asteraceae)

**DOI:** 10.12688/wellcomeopenres.27528.1

**Published:** 2026-09-16

**Authors:** Maarten J. M. Christenhusz, Sahr Mian, Royal Botanic Gardens Kew Genome Acquisition Lab, Plant Genome Sizing Collective, Darwin Tree of Life Barcoding collective, Wellcome Sanger Institute Tree of Life Management, Samples and Laboratory team, Wellcome Sanger Institute Scientific Operations: Sequencing Operations, Wellcome Sanger Institute Tree of Life Core Informatics team, Tree of Life Core Informatics collective, Darwin Tree of Life Consortium

**Affiliations:** 1 Royal Botanic Gardens Kew, Richmond, England, UK; 2 Botanical Society of Britain and Ireland, St Albans, Hertfordshire, England, UK

**Keywords:** Galatella linosyris, Goldilocks Aster, genome sequence, chromosomal, Asterales

## Abstract

We present a genome assembly of *Galatella linosyris* (Goldilocks Aster; Streptophyta; Magnoliopsida; Asterales; Asteraceae). The assembly consists of two haplotypes with total lengths of 4,455.90 megabases and 4,428.67 megabases. Most of haplotype 1 (99.26%) is scaffolded into 9 chromosomal pseudomolecules. Haplotype 2 was assembled to scaffold level. The mitochondrial sequences have lengths of 195.56 and 60.14 kilobases and the plastid genome assembly has a length of 153.44 kilobases. Gene annotation of this assembly on Ensembl identified 31,962 protein-coding genes. This assembly was generated as part of the Darwin Tree of Life project, which produces reference genomes for eukaryotic species found in Britain and Ireland.

## Species taxonomy

Eukaryota; Viridiplantae; Streptophyta; Streptophytina; Embryophyta; Tracheophyta; Euphyllophyta; Spermatophyta; Magnoliopsida; Mesangiospermae; eudicotyledons; Gunneridae; Pentapetalae; asterids; campanulids; Asterales; Asteraceae; Asteroideae; Astereae; Australasian lineages; Asterinae; *Galatella*; *Galatella linosyris* (L. Rchb.f., 1853) (NCBI:txid1155252).

## Background

*Galatella linosyris* (L.) Rchb.f. (Goldilocks Aster) is a native, lowland perennial herb of open limestone habitats. In Britain, it grows on shallow soils on limestone sea-cliffs and rocky slopes, and in cliff-top grassland and wind-pruned heath. It is a poor competitor and is usually intolerant of heavy grazing, although in Pembrokeshire it also occurs in short, sheep-grazed cliff-top grassland and heath ( Halliday *et al.*, 2023).

The species appears to be self-incompatible, and some small populations may consist of a single self-sterile clone that persists vegetatively. Plants in cultivation can spread vigorously when free from competition ( Halliday *et al.*, 2023). The species had been considered extinct in Cumbria since 2007, but in 2023 the Back on Our Map (BOOM) project reintroduced 185 plants to Humphrey Head ( Kent, 2023).

We sequenced the genome of *Galatella linosyris* as part of the Darwin Tree of Life Project, which aims to generate high-quality reference genomes for all named eukaryotic species in Britain and Ireland to support research, conservation, and the sustainable use of biodiversity. The sequenced specimen was collected from Royal Botanic Gardens Kew, Richmond, UK ( Figure 1).

**Figure 1.**
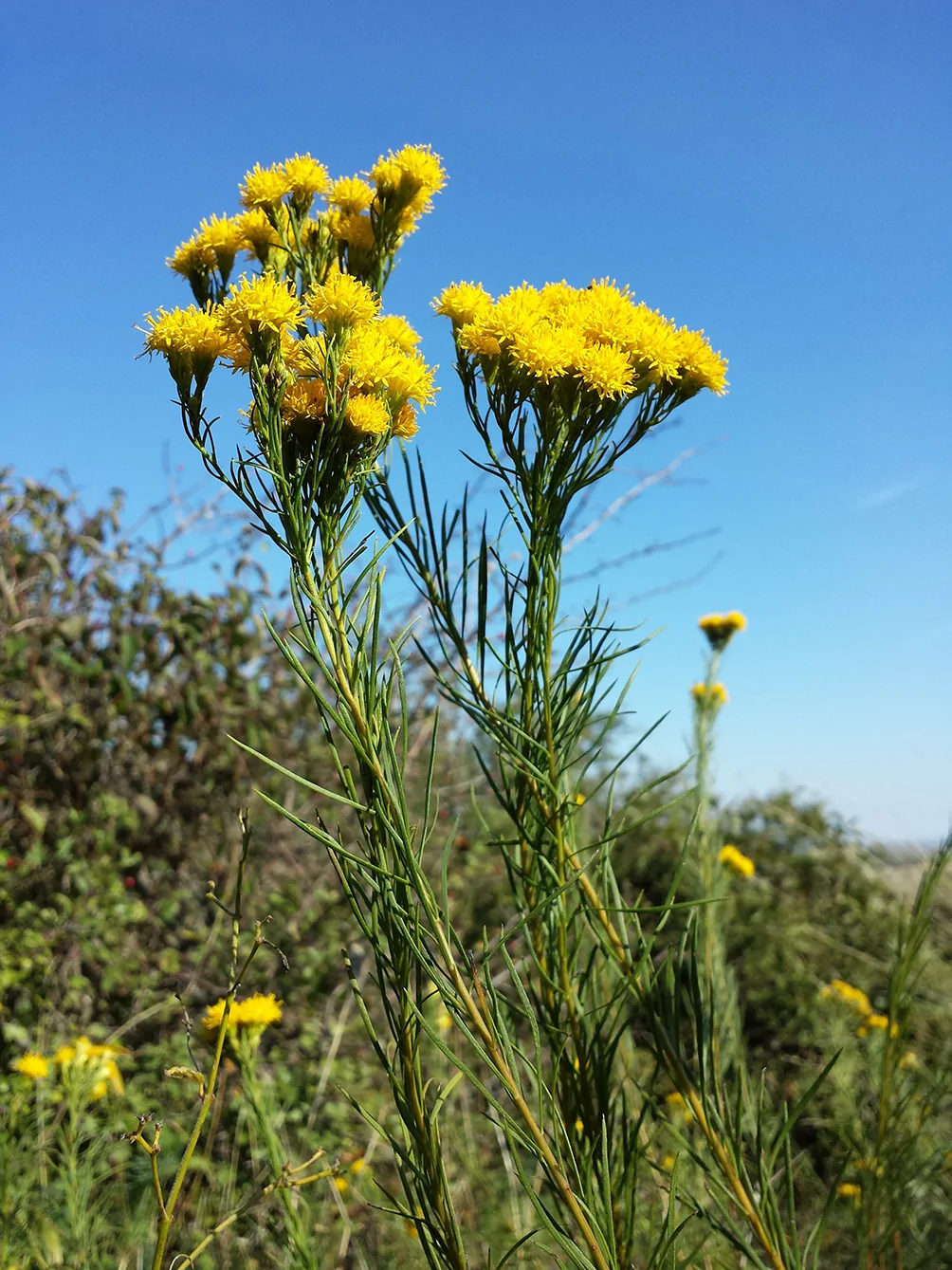
Photograph of *Galatella linosyris* by Stefan Lefnaer.

## Methods

### Sample acquisition, flow cytometry and DNA barcoding

A specimen of *Galatella linosyris* (specimen ID KDTOL10680, ToLID daGalLino1; Figure 1) was used for genome sequencing. It was collected from Royal Botanic Gardens Kew, Richmond, UK (latitude 51.4825, longitude −0.2876) on 2022-09-29. The specimen was collected by Maarten Christenhusz and Sahr Mian and identified by Maarten Christenhusz. The daGalLino1 specimen was also used for Hi-C and RNA sequencing. The herbarium voucher associated with the sequenced plant is MC9447 and is deposited in the herbarium of RBG Kew (K) K001527050.

The genome size was estimated by flow cytometry following the ‘one-step’ method outlined in Pellicer *et al*. (2021) and using propidium iodide as the fluorochrome. The General Purpose Buffer (GPB) supplemented with 3% PVP was used for isolation of nuclei ( Loureiro *et al.*, 2007), and the internal calibration standard was *Petroselinum crispum* ‘Champion Moss Curled’ with an assumed 1C-value of 2 200 Mb ( Obermayer *et al.*, 2002).

The initial identification was verified by an additional DNA barcoding process according to the framework developed by Twyford *et al*. (2024). Part of the plant specimen was preserved in silica gel desiccant ( Chase & Hills, 1991). DNA extracted from the dried plant was amplified by PCR for standard barcode markers, with the amplicons sequenced and compared to public sequence databases including GenBank and the Barcode of Life Database (BOLD) ( Ratnasingham & Hebert, 2007). Following whole genome sequence generation, the relevant DNA barcode region was also used alongside the initial barcoding data for sample tracking at the WSI ( Twyford *et al.*, 2024). The standard operating procedures for Darwin Tree of Life barcoding are available on protocols.io.

### Nucleic acid extraction

High molecular weight (HMW) DNA extraction protocols developed by the Tree of Life Core Laboratory at the Wellcome Sanger Institute (WSI) are available on protocols.io ( Howard *et al.*, 2025). Tissue from the daGalLino1 sample was weighed and triaged to select the appropriate extraction workflow.

HMW DNA was prepared for three PacBio HiFi libraries: DTOL13960909, DTOL14809403 and DTOL13700589. For DTOL13960909 and DTOL14809403, 52.0 and 57.0 mg aliquots, respectively, of the daGalLino1 leaf sample were homogenised by cryogenic bead beating. HMW DNA was extracted using the Automated Plant MagAttract v4 protocol and fragmented using the Megaruptor3 protocol for low-input PacBio sequencing. Fragmented DNA for DTOL13960909 was purified by manual SPRI, whereas DNA for DTOL14809403 was purified by automated SPRI.

For DTOL13700589, a 57.0 mg aliquot of the daGalLino1 leaf sample was homogenised using Covaris cryoPREP. HMW DNA was extracted using the recorded MagAttract Standard 48xrn workflow, fragmented using the Megaruptor3 protocol for low-input PacBio sequencing, and purified by manual SPRI.

The concentration of the sheared and purified DNA was assessed using a NanoDrop spectrophotometer and Qubit Fluorometer using the Qubit dsDNA High Sensitivity Assay kit. Fragment size distribution was evaluated by running the sample on the FemtoPulse system. Post-shearing QC measurements were recorded separately for the three PacBio library preparations. For library DTOL13960909, final post-shearing DNA measurements included a Qubit concentration of 31.4 ng/μL, a yield of 1,475.80 ng, and an average fragment size of 16.8 kb. The 260/280 spectrophotometric ratio was 1.91, and the 260/230 ratio was 2.78. The Genomic Quality Number (GQN) was 8.2. For library DTOL14809403, final post-shearing DNA measurements included a Qubit concentration of 95.6 ng/μL, a yield of 5,258.00 ng, and an average fragment size of 18.1 kb. The 260/280 spectrophotometric ratio was 1.83, and the 260/230 ratio was 2.01. The Genomic Quality Number (GQN) was 7.7. For library DTOL13700589, final post-shearing DNA measurements included a Qubit concentration of 20.79 ng/μL, a yield of 977.13 ng, and an average fragment size of 17.9 kb. The 260/280 spectrophotometric ratio was 1.78, and the 260/230 ratio was 1.45. The Genomic Quality Number (GQN) was 8.8.

RNA was extracted from leaf tissue of daGalLino1 in the Tree of Life Laboratory at the WSI using the RNA Extraction: Automated MagMax™ *mir*Vana protocol. The RNA concentration was assessed using a NanoDrop spectrophotometer and a Qubit Fluorometer using the Qubit RNA Broad-Range Assay kit. Analysis of the integrity of the RNA was done using the Agilent RNA 6000 Pico Kit and Eukaryotic Total RNA assay.

### PacBio HiFi library preparation and sequencing

Library preparation and sequencing were performed at the WSI Scientific Operations core. Libraries DTOL13960909, DTOL14809403 and DTOL13700589 were prepared using the SMRTbell Prep Kit 3.0 (Pacific Biosciences) according to the manufacturer’s instructions. The kit includes reagents for end repair/A-tailing, adapter ligation, post-ligation SMRTbell bead clean-up, and nuclease treatment. Size selection and clean-up were performed using diluted AMPure PB beads (Pacific Biosciences). DNA concentration was quantified using a Qubit Fluorometer v4.0 (Thermo Fisher Scientific) and the Qubit 1X dsDNA HS assay kit. Final library fragment size was assessed with the Agilent Femto Pulse Automated Pulsed Field CE Instrument (Agilent Technologies) using the gDNA 55 kb BAC analysis kit.

Libraries DTOL13960909 and DTOL14809403 were sequenced in three runs on a Revio instrument (Pacific Biosciences, California, USA). Prepared libraries with a molarity greater than 2 nM were normalised to 2 nM, and 15 μL was used for making complexes. For libraries with a molarity below 2 nM, the available 10 μL library volume was used without normalisation. Primers were annealed and polymerases were bound to generate circularised complexes, following the manufacturer’s instructions. Complexes were purified using 1.2× SMRTbell beads, diluted to the Revio loading concentration of 200–300 pM, and spiked with a Revio sequencing internal control. Sequencing used Revio 25 M SMRT cells. SMRT Link software (Pacific Biosciences), a web-based workflow manager, was used to configure and monitor the runs and to carry out primary and secondary data analysis.

Library DTOL13700589 was sequenced using the Sequel IIe system (Pacific Biosciences, California, USA). The concentration of the library loaded onto the Sequel system was in the range 40–135 pM. SMRT Link software, a PacBio web-based workflow manager, was used to set up and monitor the run, and to perform primary and secondary analysis of the data upon completion.

### Hi-C

***Sample preparation and crosslinking***

Hi-C data were generated from 57 mg of the daGalLino1 leaf sample using the Arima-HiC v2 kit (Arima Genomics). Tissue was finely ground using the Covaris cryoPREP Dry Pulverizer (Covaris), and then subjected to nuclei isolation. Nuclei were isolated using a modified protocol based on the Qiagen QProteome Cell Compartment Kit (Qiagen), in which only the Lysis and CE2 buffers were used, with QIAshredder spin columns. After isolation, nuclei were fixed using formaldehyde to a final concentration of 2% to crosslink the DNA. The crosslinked DNA was then digested and biotinylated according to the manufacturer’s instructions. A clean-up step was performed with SPRIselect beads before library preparation. DNA concentration was quantified using the Qubit Fluorometer v4.0 (Thermo Fisher Scientific) and the Qubit HS Assay Kit, following the manufacturer’s instructions.

***Hi-C library preparation and sequencing***

Biotinylated DNA constructs were fragmented using a Covaris E220 sonicator and size selected to 400–600 bp using SPRISelect beads. DNA was enriched with Arima-HiC v2 kit Enrichment beads. End repair, A-tailing, and adapter ligation were carried out with the NEBNext Ultra II DNA Library Prep Kit (New England Biolabs), following a modified protocol where library preparation occurs while DNA remains bound to the Enrichment beads. Library amplification was performed using KAPA HiFi HotStart mix and a custom Unique Dual Index (UDI) barcode set (Integrated DNA Technologies). Depending on sample concentration and biotinylation percentage determined at the crosslinking stage, libraries were amplified with 10–16 PCR cycles. Post-PCR clean-up was performed with SPRISelect beads. Libraries were quantified using the AccuClear Ultra High Sensitivity dsDNA Standards Assay Kit (Biotium) and a FLUOstar Omega plate reader (BMG Labtech).

Prior to sequencing, libraries were normalised to 10 ng/μL. Normalised libraries were quantified again to create equimolar and/or weighted 2.8 nM pools. Pool concentrations were checked using the Agilent 4200 TapeStation (Agilent) with High Sensitivity D500 reagents before sequencing. Sequencing was performed using paired-end 150 bp reads on the Illumina NovaSeq 6000.

### RNA library preparation and sequencing

Libraries were prepared using the NEBNext® Ultra II Directional RNA Library Prep Kit for Illumina (New England Biolabs), following the manufacturer’s instructions. Poly(A) mRNA was isolated from 100 ng of total RNA using oligo (dT) beads, converted to cDNA, and uniquely indexed; 14 PCR cycles were performed. Libraries were prepared with an intended average fragment size of 100–300 bp. Libraries were quantified, normalised, and pooled equimolarly to a final concentration of 2.8 nM before dilution to 150 pM for loading. Sequencing was carried out on the Illumina NovaSeq X, generating paired-end reads.

### Genome assembly

Prior to assembly of the PacBio HiFi reads, a database of *k*-mer counts ( *k* = 31) was generated from the filtered reads using FastK. GenomeScope2 ( Ranallo-Benavidez *et al.*, 2020) was used to analyse the *k*-mer frequency distributions, providing estimates of genome size, heterozygosity, and repeat content.

The HiFi reads were assembled using Hifiasm in Hi-C phasing mode ( Cheng *et al.*, 2021, 2022), producing two haplotypes. Hi-C reads ( Rao *et al.*, 2014) were mapped to the primary contigs using bwa-mem2 ( Vasimuddin *et al.*, 2019). Contigs were further scaffolded with Hi-C data in YaHS ( Zhou *et al.*, 2023), using the --break option for handling potential misassemblies. The scaffolded assemblies were evaluated using Gfastats ( Formenti *et al.*, 2022), BUSCO ( Manni *et al.*, 2021) and MerquryFK ( Rhie *et al.*, 2020). The organelle genomes were assembled using OATK ( Zhou *et al.*, 2025). The comparator for the mitochondrial assembly was the mitochondrial genome of *Aster batangensis* (NC_082289.1).

### Assembly curation

The assembly was decontaminated using the Assembly Screen for Cobionts and Contaminants ( ASCC) pipeline. TreeVal was used to generate the flat files and maps for use in curation. Manual curation was conducted primarily in PretextView and HiGlass ( Kerpedjiev *et al.*, 2018). Scaffolds were visually inspected and corrected as described by Howe *et al*. (2021). Manual corrections included 19 breaks and 39 joins, which reduced the scaffold count by 3.0%. The curation process is described at sanger-tol/curation-resources . PretextSnapshot was used to generate a Hi-C contact map of the final assembly.

### Assembly quality assessment

The MerquryFK tool ( Rhie *et al.*, 2020) was run in a Singularity container ( Kurtzer *et al.*, 2017) to evaluate *k*-mer completeness and assembly quality for both haplotypes using the *k*-mer database ( *k* = 31) computed prior to genome assembly. The analysis outputs included assembly QV scores and completeness statistics.

The genome was analysed using the BlobToolKit pipeline, a Nextflow implementation of the earlier Snakemake version ( Challis *et al.*, 2020). PacBio reads were aligned to the assembly using minimap2 ( Li, 2018) and processed with SAMtools ( Danecek *et al.*, 2021) to generate coverage tracks. The pipeline runs BUSCO ( Manni *et al.*, 2021) using lineages identified from the NCBI Taxonomy ( Schoch *et al.*, 2020). For the three basal BUSCO lineages, eukaryota, bacteria and archaea, BUSCO genes are aligned to the UniProt Reference Proteomes database ( Bateman *et al.*, 2023) using DIAMOND BLASTp ( Buchfink *et al.*, 2021). The genome assembly is divided into chunks based on the density of BUSCO genes from the closest taxonomic lineage, and each chunk is aligned to the UniProt Reference Proteomes database using DIAMOND BLASTx. Sequences without DIAMOND BLASTx hits are extracted using seqtk and aligned to the NT database using BLASTn ( Altschul *et al.*, 1990). The outputs are consolidated into a BlobDir for visualisation in BlobToolKit. The BlobToolKit pipeline was developed using nf-core tooling ( Ewels *et al.*, 2020) and MultiQC ( Ewels *et al.*, 2016), with containerisation through Docker ( Merkel, 2014) and Singularity ( Kurtzer *et al.*, 2017).

## Genome sequence report

### Sequence data

The genome of a specimen of *Galatella linosyris* was sequenced using Pacific Biosciences single-molecule HiFi long reads, generating 234.14 Gb (gigabases) from 22.01 million reads, which were used to assemble the genome. GenomeScope2.0 analysis using a *p* = 2 model gave a *k*-mer-based genome size estimate of 4,461.12 Mb, with a heterozygosity of 0.33% and repeat content of 64.93% ( Figure 2). Using flow cytometry, the genome size (1C-value) of the sample was estimated to be 4.99 pg, equivalent to 4,880.00 Mb. These estimates guided expectations for the assembly. Based on the estimated genome size, the sequencing data provided approximately 51× coverage. Hi-C sequencing produced 939.74 Gb from 3,111.74 million read pairs, which were used to scaffold the assembly. RNA sequencing data were also generated and are available in public sequence repositories. Table 1 summarises the specimen and sequencing details.

**Figure 2.**
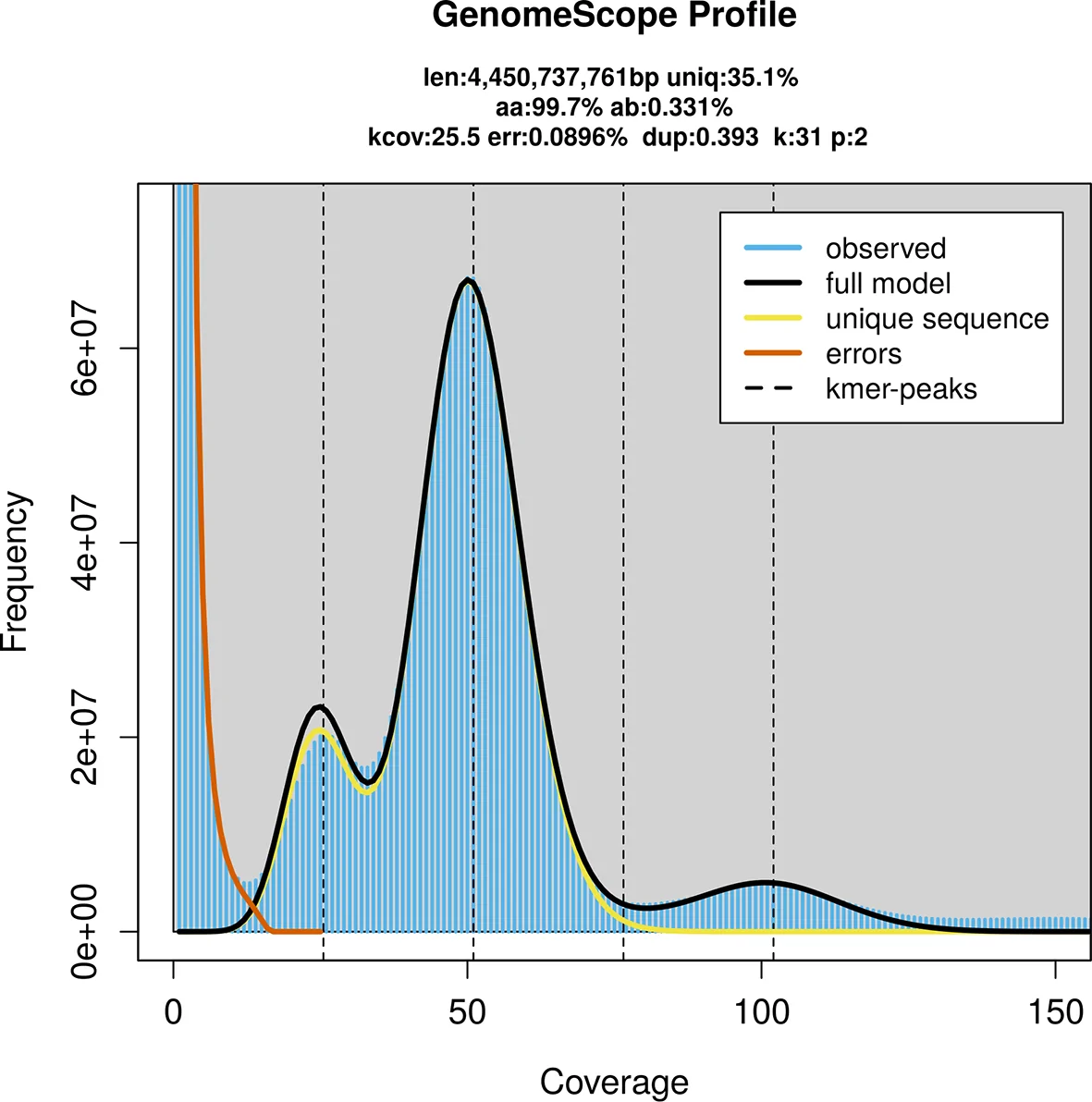
Frequency distribution of *k*-mers generated using GenomeScope2. The plot shows observed and modelled *k*-mer spectra, providing estimates of genome size, heterozygosity, and repeat content based on unassembled sequencing reads.

**Table 1.** Specimen and sequencing data for *Galatella linosyris* (BioProject PRJEB80058).

Platform	PacBio HiFi	Hi-C	RNA-seq
**ToLID**	daGalLino1	daGalLino1	daGalLino1
**Specimen ID**	KDTOL10680	KDTOL10680	KDTOL10680
**BioSample (source individual)**	SAMEA112290144	SAMEA112290144	SAMEA112290144
**BioSample (tissue)**	SAMEA112290364	SAMEA112290364	SAMEA112290364
**Tissue**	leaf	leaf	leaf
**Instrument**	Revio (3 runs); Sequel IIe (1 run)	Illumina NovaSeq 6000	Illumina NovaSeq X
**Run accessions**	ERR13660076; ERR13660077; ERR13660079; ERR13660078	ERR13670040	ERR15551477
**Read count total**	22.01 million reads	3,111.74 million read pairs	48.96 million read pairs
**Base count total**	234.14 Gb	939.74 Gb	14.79 Gb

### Assembly statistics

The genome was assembled into two haplotypes using Hi-C phasing. Haplotype 1 was curated to chromosome level, while haplotype 2 was assembled to scaffold level. The final assembly has a total length of 4,455.90 Mb in 456 scaffolds, with 2,215 gaps, and a scaffold N50 of 521.52 Mb ( Table 2).

**Table 2.** Genome assembly statistics for *Galatella linosyris.*

Genome assembly	Haplotype 1	Haplotype 2
**Assembly name**	daGalLino1.hap1.1	daGalLino1.hap2.1
**Assembly accession**	GCA_965198375.1	GCA_965198385.1
**Assembly level**	chromosome	scaffold
**Span (Mb)**	4,455.90	4,428.67
**Number of chromosomes**	9	-
**Number of contigs**	2,671	2,556
**Contig N50 (Mb)**	3.13	3.14
**Number of scaffolds**	456	388
**Scaffold N50 (Mb)**	521.52	514.59
**Longest scaffold length (Mb)**	641.89	634.06
**Organelles**	Mitochondrial genomes: 195.56 and 60.14 kb; Plastid genome: 153.44 kb	-

Most of the assembly sequence (99.26%) was assigned to 9 chromosomal-level scaffolds. These chromosome-level scaffolds, confirmed by Hi-C data, are named according to size ( Figure 3; Table 3).

**Figure 3.**
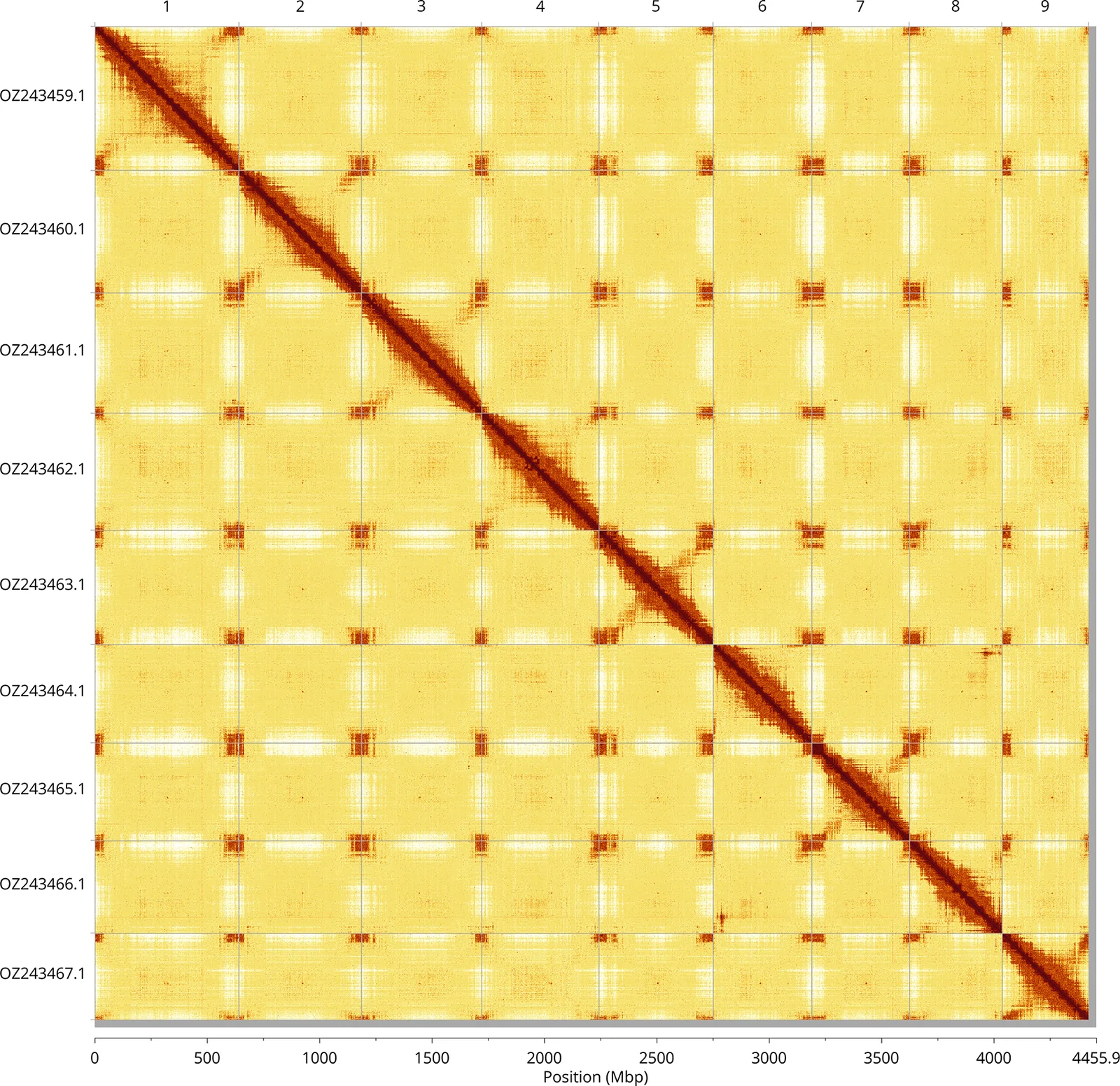
Hi-C contact map of the *Galatella linosyris* genome assembly. Assembled chromosomes are shown in order of size and labelled along the axes, with a megabase scale shown below. The plot was generated using PretextSnapshot.

**Table 3.** Chromosomal pseudomolecules in the haplotype 1 genome assembly of *Galatella linosyris* daGalLino1.

INSDC accession	Molecule	Length (Mb)	GC%
OZ243459.1	1	641.89	36
OZ243460.1	2	544.65	36
OZ243461.1	3	535.62	36
OZ243462.1	4	521.52	36
OZ243463.1	5	508.48	36
OZ243464.1	6	438.03	36
OZ243465.1	7	434.80	36
OZ243466.1	8	411.94	36
OZ243467.1	9	386.04	36

Two mitochondrial genomes (lengths 195.56 kb [OZ243468.1] and 60.14 kb [OZ243469.1]) and the plastid genome (length 153.44 kb, OZ243470.1) were also assembled. These sequences are included as contigs in the multifasta file of the genome submission and as standalone records.

### Assembly quality metrics

For haplotype 1, the estimated QV is 68.4, and for haplotype 2, 68.4. When the two haplotypes are combined, the assembly achieves an estimated QV of 68.4. The *k*-mer completeness is 92.53% for haplotype 1, 92.12% for haplotype 2, and 98.49% for the combined haplotypes ( Figure 4).

**Figure 4.**
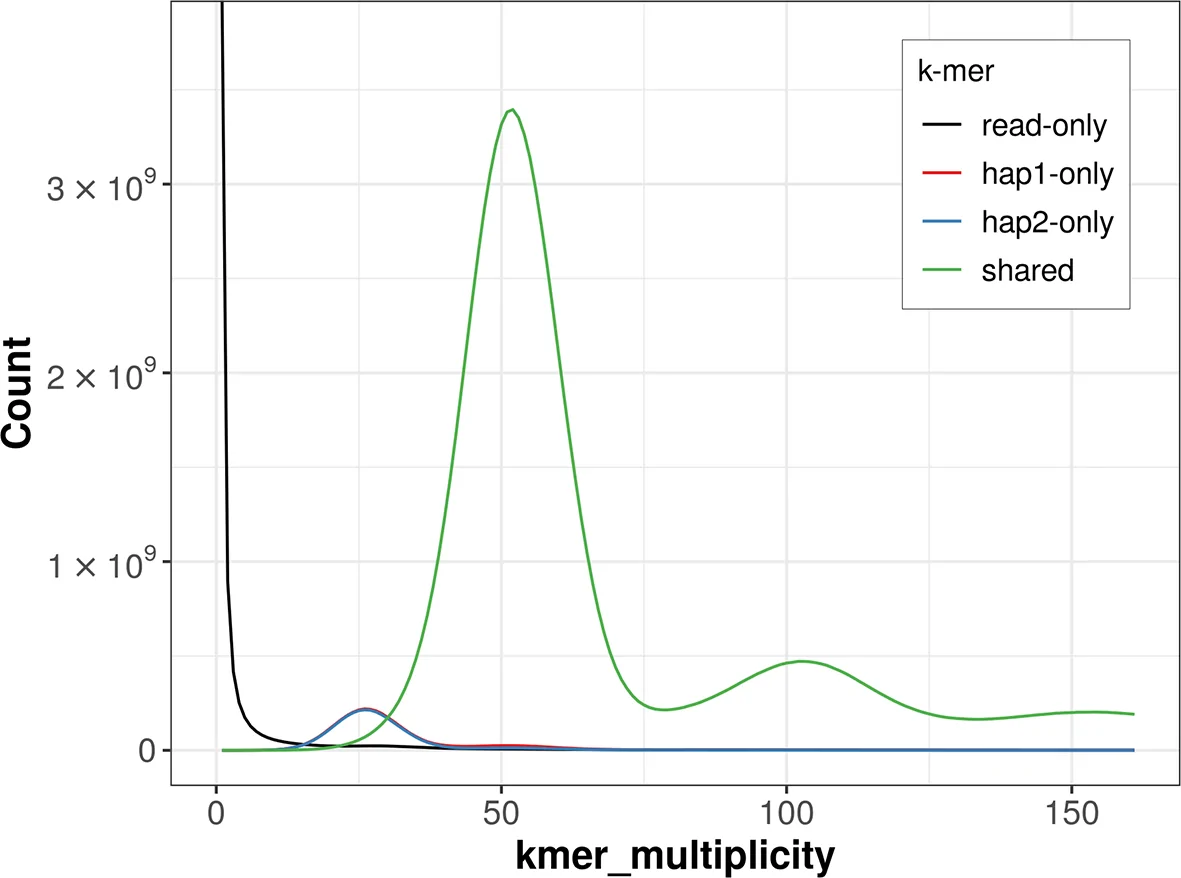
Evaluation of *k*-mer completeness using MerquryFK. This plot illustrates the recovery of *k*-mers from the original read data in the final assemblies. The horizontal axis represents *k*-mer multiplicity, and the vertical axis shows the number of *k*-mers. The black curve represents *k*-mers that appear in the reads but are not assembled. The green curve corresponds to *k*-mers shared by both haplotypes, and the red and blue curves show *k*-mers found only in one of the haplotypes.

BUSCO analysis using the eudicots_odb10 reference set ( *n* = 2,326) identified 97.2% of the expected gene set (single = 88.3%, duplicated = 8.9%) for haplotype 1. The snail plot in Figure 5 summarises the scaffold length distribution and other assembly statistics for haplotype 1. The blob plot in Figure 6 shows the distribution of scaffolds by GC proportion and coverage for haplotype 1.

**Figure 5.**
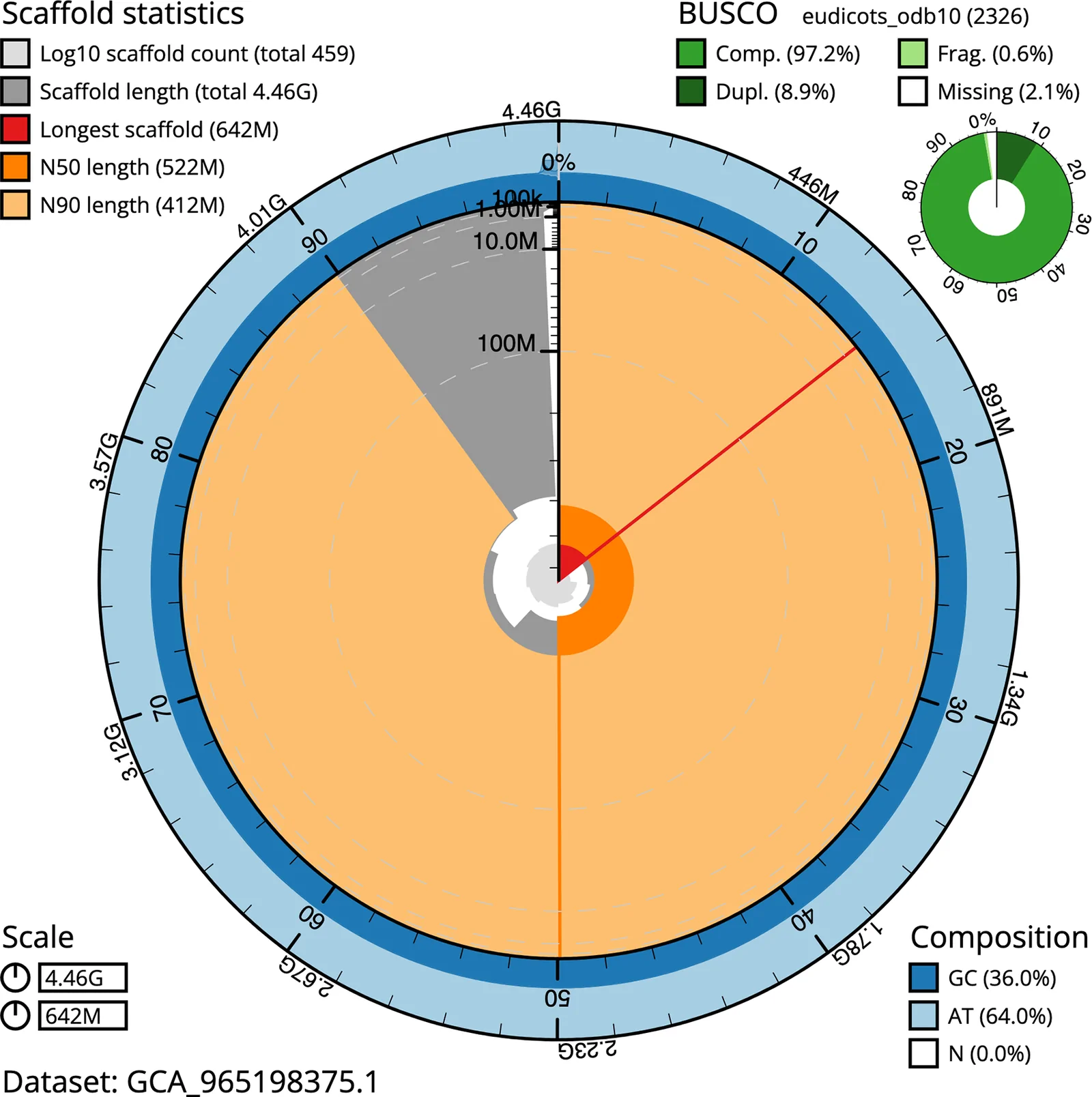
Assembly metrics for daGalLino1.hap1.1. The BlobToolKit snail plot provides an overview of assembly metrics and BUSCO gene completeness. The circumference represents the length of the whole genome sequence, and the main plot is divided into 1 000 bins around the circumference. The outermost blue tracks display the distribution of GC, AT, and N percentages across the bins. Scaffolds are arranged clockwise from longest to shortest and are depicted in dark grey. The longest scaffold is indicated by the red arc, and the deeper orange and pale orange arcs represent the N50 and N90 lengths. A light grey spiral at the centre shows the cumulative scaffold count on a logarithmic scale. A summary of complete, fragmented, duplicated, and missing BUSCO genes in the eudicots_odb10 set is presented at the top right. An interactive version of this figure can be accessed on the BlobToolKit viewer.

**Figure 6.**
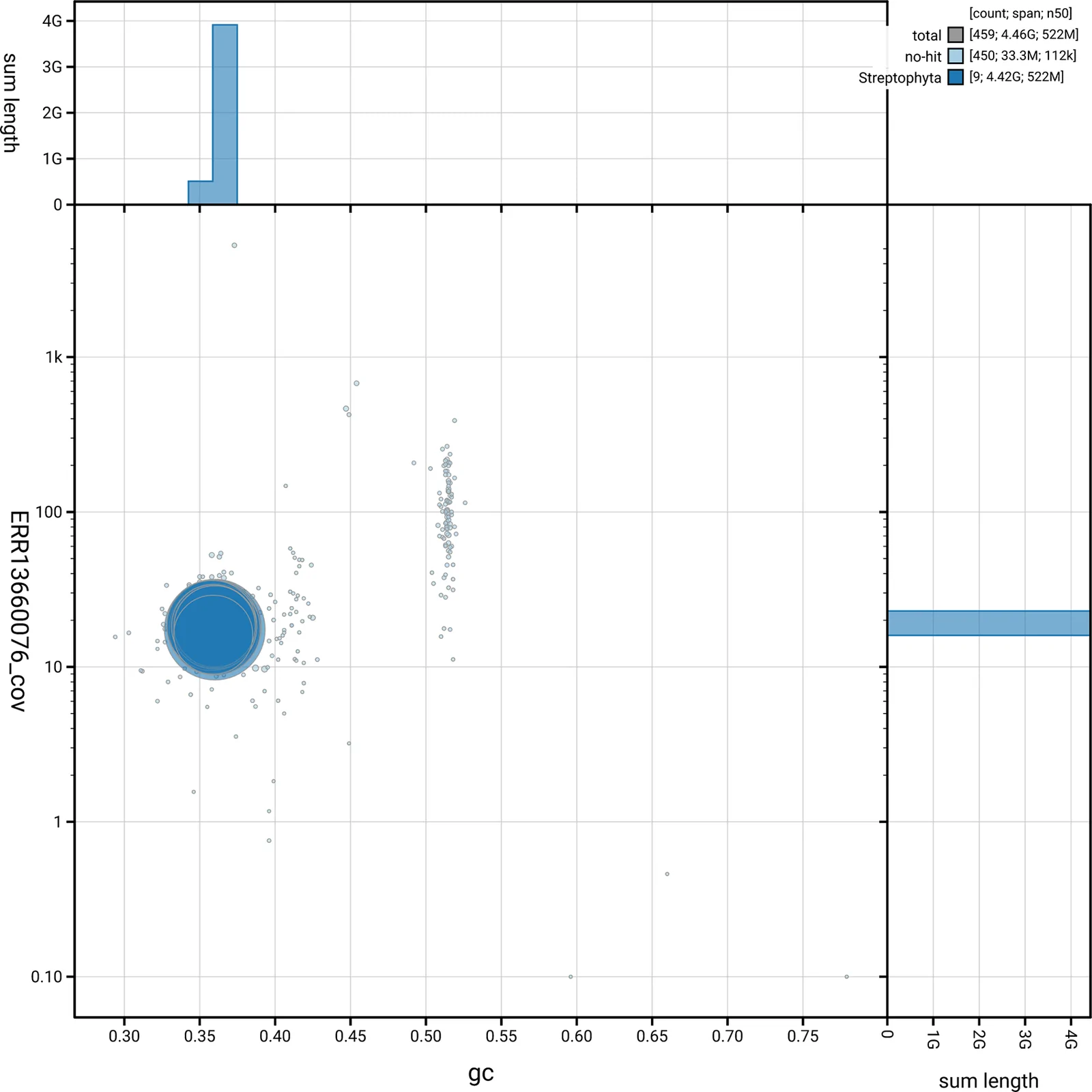
BlobToolKit blob plot for daGalLino1.hap1.1. The plot shows base coverage (vertical axis) and GC content (horizontal axis). The circles represent scaffolds, with the size proportional to scaffold length and the colour representing phylum membership. The histograms along the axes display the total length of sequences distributed across different levels of coverage and GC content. An interactive version of this figure is available on the BlobToolKit viewer.

Table 4 lists the assembly metric benchmarks adapted from Rhie *et al.* (2021) and the Earth BioGenome Project (EBP) Report on Assembly Standards. The EBP metric calculated for haplotype 1 is **6.C.Q68**, meeting the recommended 6.C.Q40 reference standard.

**Table 4.** Earth Biogenome Project summary metrics for the *Galatella linosyris* assembly.

Measure	Value	Benchmark
EBP summary (haplotype 1)	6.C.Q68	6.C.Q40
Contig N50 length	3.13 Mb	≥ 1 Mb
Scaffold N50 length	521.52 Mb	= chromosome N50
Consensus quality (QV)	Haplotype 1: 68.4; haplotype 2: 68.4; combined: 68.4	≥ 40
*k*-mer completeness	Haplotype 1: 92.53%; Haplotype 2: 92.12%; combined: 98.49%	> 90%
BUSCO	Haplotype 1: C:97.2% [S:88.3%, D:8.9%], F:0.6%, M:2.1%, n:2,326; Haplotype 2: C:97.1% [S:88.1%, D:9.0%], F:0.6%, M:2.3%, n:2,326	S > 90%; D < 5%
Percentage of assembly assigned to chromosomes	99.26%	≥ 90%

*Note*: The EBP summary uses log10(Contig N50); chromosome-level (C) or log10(Scaffold N50); Q (Merqury QV). BUSCO: C = complete; S = single-copy; D = duplicated; F = fragmented; M = missing; n = orthologues.

**Table 5.** Software versions and sources used for *Galatella linosyris.*

Software	Version	Source
BLAST	2.14.0	https://ftp.ncbi.nlm.nih.gov/blast/executables/blast+/
BlobToolKit	4.4.5	https://github.com/genomehubs/blobtoolkit
blobtk	0.5.1	https://github.com/genomehubs/blobtk
BUSCO	5.7.1	https://gitlab.com/ezlab/busco
bwa-mem2	2.2.1	https://github.com/bwa-mem2/bwa-mem2
DIAMOND	2.1.8	https://github.com/bbuchfink/diamond
fasta_windows	0.2.4	https://github.com/tolkit/fasta_windows
FastK	1.1	https://github.com/thegenemyers/FASTK
GenomeScope2.0	2.0.1	https://github.com/tbenavi1/genomescope2.0
Gfastats	1.3.6	https://github.com/vgl-hub/gfastats
Hifiasm	0.19.8-r603	https://github.com/chhylp123/hifiasm
HiGlass	1.13.4	https://github.com/higlass/higlass
MerquryFK	1.1.0-c1	https://github.com/thegenemyers/MERQURY.FK
Minimap2	2.24-r1122; 2.28-r1209	https://github.com/lh3/minimap2
Oatk	0.9	https://github.com/c-zhou/oatk
MultiQC	1.14; 1.17 and 1.18	https://github.com/MultiQC/MultiQC
Nextflow	23.10.1; 24.10.4	https://github.com/nextflow-io/nextflow
PretextSnapshot	0.0.6	https://github.com/sanger-tol/PretextSnapshot
PretextView	1.0.3	https://github.com/sanger-tol/PretextView
samtools	1.17; 1.18; 1.2; 1.21; 1.6	https://github.com/samtools/samtools
sanger-tol/ascc	0.1.0	https://github.com/sanger-tol/ascc
sanger-tol/blobtoolkit	v0.7.1	https://github.com/sanger-tol/blobtoolkit
sanger-tol/curationpretext	1.4.2	https://github.com/sanger-tol/curationpretext
Seqtk	1.4-r122	https://github.com/lh3/seqtk
Singularity	3.9.0	https://github.com/sylabs/singularity
TreeVal	1.1.1	https://github.com/sanger-tol/treeval
YaHS	1.2a.2	https://github.com/c-zhou/yahs

### Genome annotation report

The *Galatella linosyris* genome assembly (GCA_965198375.1) was annotated by Ensembl at the European Bioinformatics Institute (EBI). This annotation includes 53,897 transcribed mRNAs from 31,962 protein-coding and 9,944 non-coding genes. The average transcript length is 3,056.36 bp, with an average of 1.29 coding transcripts per gene and 4.64 exons per transcript. For further information about the annotation, please refer to the annotation page on Ensembl.

## Author information

• Members of the Royal Botanic Gardens Kew Genome Acquisition Lab
• Members of the Plant Genome Sizing Collective
• Members of the Darwin Tree of Life Barcoding collective
• Members of the Wellcome Sanger Institute Tree of Life Management, Samples and Laboratory team
• Members of Wellcome Sanger Institute Scientific Operations – Sequencing Operations
• Members of the Wellcome Sanger Institute Tree of Life Core Informatics team
• Members of the Tree of Life Core Informatics collective
• Members of the Darwin Tree of Life Consortium

## Wellcome sanger institute – legal and governance

The materials that have contributed to this genome note have been supplied by a Darwin Tree of Life Partner. The submission of materials by a Darwin Tree of Life Partner is subject to the **‘Darwin Tree of Life Project Sampling Code of Practice’**, which can be found in full on the Darwin Tree of Life website. By agreeing with and signing up to the Sampling Code of Practice, the Darwin Tree of Life Partner agrees they will meet the legal and ethical requirements and standards set out within this document in respect of all samples acquired for, and supplied to, the Darwin Tree of Life Project. Further, the Wellcome Sanger Institute employs a process whereby due diligence is carried out proportionate to the nature of the materials themselves, and the circumstances under which they have been/are to be collected and provided for use. The purpose of this is to address and mitigate any potential legal and/or ethical implications of receipt and use of the materials as part of the research project, and to ensure that in doing so we align with best practice wherever possible. The overarching areas of consideration are:

• Ethical review of provenance and sourcing of the material
• Legality of collection, transfer and use (national and international)

Each transfer of samples is further undertaken according to a Research Collaboration Agreement or Material Transfer Agreement entered into by the Darwin Tree of Life Partner, Genome Research Limited (operating as the Wellcome Sanger Institute), and in some circumstances, other Darwin Tree of Life collaborators.

## Data Availability

European Nucleotide Archive: Galatella linosyris. Accession number PRJEB80058; https://identifiers.org/ena.embl/PRJEB80058. The genome sequence is released openly for reuse. The *Galatella linosyris* genome sequencing initiative is part of the Darwin Tree of Life Project ( PRJEB40665) and Sanger Institute Tree of Life Programme ( PRJEB43745). All raw sequence data and the assembly have been deposited in INSDC databases. Raw data and assembly accession identifiers are reported in Tables 1 and 2. Pipelines used for genome assembly at the WSI Tree of Life are available at https://pipelines.tol.sanger.ac.uk/pipelines. Table 5 lists software versions used in this study.
