# Supplementary material for: The genome sequence of Goldilocks Aster, Galatella linosyris (L. Rchb.f., 1853) (Asterales: Asteraceae)
Source: Wellcome Open Res. 2026 Sep 16;11:678. [Version 1] doi: 10.12688/wellcomeopenres.27528.1 (PMC13684020; doi:10.12688/wellcomeopenres.27528.1)
Supplement: Peer review history

# Reviewer report: Reviewer response for version 1

**DOI:** 10.21956/wellcomeopenres.30299.r171424

**Published:** 2026-10-20

**Authors:** Rong Zhang

**Affiliations:** 1 Kunming Institute of Botany Chinese Academy of Sciences (Ringgold ID: 26445), Kunming, Yunnan, China

This study provides a useful chromosome-level genome resource for *Galatella linosyris*. The sequencing and assembly quality generally meet current standards for chromosome-scale genome assemblies in large-scale biodiversity genomics projects. Please clearly specify the plant tissues used for DNA and RNA extraction (e.g., young leaves) in the Methods section. In addition, please provide brief background information on the chromosome number and ploidy status of *G. linosyris* in the Background section.

Are sufficient details of methods and materials provided to allow replication by others?

Yes

Is the rationale for creating the dataset(s) clearly described?

Yes

Are the datasets clearly presented in a useable and accessible format?

Yes

Are the protocols appropriate and is the work technically sound?

Yes

Reviewer Expertise:

Phylogeny, phylogenomics, and evolution

I confirm that I have read this submission and believe that I have an appropriate level of expertise to confirm that it is of an acceptable scientific standard.

# Reviewer report: Reviewer response for version 1

**DOI:** 10.21956/wellcomeopenres.30299.r168942

**Published:** 2026-10-10

**Authors:** Matthew W. Davis

**Affiliations:** 1 University of California, California, USA

**Article summary:**

 The article reports a genome assembly of Galatella linosyris, an Aster native to Britain's open limestone habitats. The species is self-incompatible and sometimes forms small populations of clonal stands. The authors assembled a reference genome using the current best practices for data generation and genome assembly, and the manuscript complies with the general Tree of Life format. Overall, the assembled genome appears to be of high quality.

**Lines with comments and questions:**

 “...the relevant DNA barcode region was also used alongside the initial barcoding data for sample tracking at the WSI (Twyford et al., 2024).”

- Wellcome Sanger Institute should be fully written here, as it is the first mention of the abbreviation. This is a common issue with the Tree of Life format publications that should be addressed so that it stops propagating with new entries. 

“For further information about the annotation, please refer to the annotation page on Ensembl.”

- The annotation page link is actually a link to download the GTF file. This should be worded differently, or actually link to the Ensembl page from which the data can be downloaded.

**Final Reviewer thoughts:**

 The genome appears to be of high quality and the figures are consistent with a strong assembly. From the frequency distribution of k-mers and the percentage of duplicated BUSCO genes, there may have been an ancient whole genome duplication that would be interesting to explore in future work. The authors provided a high quality assembly and I found little to raise concerns about.

Are sufficient details of methods and materials provided to allow replication by others?

Yes

Is the rationale for creating the dataset(s) clearly described?

Yes

Are the datasets clearly presented in a useable and accessible format?

Yes

Are the protocols appropriate and is the work technically sound?

Yes

Reviewer Expertise:

Plant genomics, genetics, genome assembly

I confirm that I have read this submission and believe that I have an appropriate level of expertise to confirm that it is of an acceptable scientific standard.
